# Chronic kidney disease and pregnancy outcomes

**DOI:** 10.1038/s41598-021-00670-3

**Published:** 2021-10-29

**Authors:** Jan Dvořák, Michal Koucký, Eva Jančová, Marek Mysliveček, Vladimír Tesař, Antonín Pařízek

**Affiliations:** 1 Department of Obstetrics and Gynaecology, General University Hospital in Prague and First Faculty of Medicine, Charles University, Apolinářská 18, 128 08 Prague 2, Czech Republic; 2 Department of Nephrology, General University Hospital in Prague and First Faculty of Medicine, Charles University, U Nemocnice 499/2, 128 08 Prague 2, Czech Republic

**Keywords:** Predictive markers, Chronic kidney disease

## Abstract

Pregnancy complicated by CKD is currently not fully understood topic. Outcome of pregnancy in patients with CKD is related to impaired glomerular filtration rate and the degree of proteinuria. In our study we evaluated the association of serum creatinine level and proteinuria with both maternal and fetal outcomes in the cohort of 84 pregnant patients with CKD. In CKD group we confirmed negative correlation of highest serum creatinine level in pregnancy to fetal weight (*p* value < 0.001) and gestation period (*p* value < 0.001). Likewise, negative correlation of preconception serum creatinine to fetal weight (*p* value < 0.001) and gestation period (*p* value 0.002). Negative correlation of proteinuria to gestation period (*p* value < 0.001) and fetal weight (*p* value < 0.001) was also demonstrated. CKD is serious risk factor for pregnancy outcome. Proteinuria and serum creatinine level should be examined before pregnancy and regularly monitored during pregnancy. Higher serum creatinine levels and higher proteinuria predispose to shorter gestation period and lower birth weight of the neonate.

## Introduction

With increasing prevalence of chronic kidney disease (CKD) in general population, female patients in fertile age with impaired kidney function are becoming more common. The presence of CKD in pregnant patients has been associated with poorer pregnancy outcomes.

CKD is defined as abnormalities of kidney structure or function, present for more than 3 months, with implications for health. Besides the obvious impairment of kidney excretory function by means of decreased glomerular filtration rate (GFR), the term CKD also includes other kidney abnormalities, such as proteinuria, tubular disorders and structural/ congenital abnormalities^1^.

Female patients with advanced CKD have lower fertility rate, but they can and often do get pregnant. Cases of pregnancies in patients with end-stage kidney disease (ESKD) also exist. The prevalence of mild to moderate CKD (CKD 1–3) in pregnant patients is however unknown^2^.

The spectrum of kidney disorders in female fertile patients is broad. The most frequent would be congenital abnormalities of the kidney and urinary tract, hypertensive nephrosclerosis and diabetic nephropathy. Also, certain glomerulopathies are quite common, such as IgA nephropathy and lupus nephritis (LN).

Pregnant patients with underlying CKD have been reported to be at increased risk of preterm delivery, superimposed preeclampsia and have higher rate of fetal growth restriction (FGR). Patients with mild CKD (CKD 1–2) usually have favorable post-partum renal outcomes. Significant decrease of kidney functions during pregnancy is, however, observed in patients with more severe kidney dysfunction (CKD 3b-5) already at conception^3–5^.

Decreased GFR and increased urinary protein excretion are both most commonly used and defined markers of kidney damage. Estimated GFR calculations are not to be used in pregnant population, as they inconsistently underestimate renal function. Serum creatinine concentration, therefore, remains the only standard assessment for kidney function in these patients. Serum creatinine of > 77 μmol/l (0.87 mg/dl) should be considered upper limit for pregnancy^6^. Urinary protein excretion increases in normal pregnancy from less than 150 mg/day in non-pregnant individuals to up to 300 mg/day in pregnancy (protein/creatinine ratio up to 0.3 mg/mg)^7,8^.

The aim of our study was to assess the pregnancy outcomes in patients with CKD.

## Patient characteristics and methods

We have established a retrospective cohort of 84 female CKD patients, who delivered living new-born between the years 2004–2019 at the Department of Obstetrics and Gynecology, General University Hospital in Prague. All patients were followed by nephrologist before conception. All procedures performed in our study were in accordance with the ethical standards of the General university hospital in Prague Ethics committee and with the 1964 Helsinki declaration and its later amendments. All patients signed informed consent regarding analysis and publication of collected anonymized data. Two values of both serum creatinine (μmol/l) and proteinuria (g/24 h) were recorded—preconception level (last known value before pregnancy) and maximal (the highest value during pregnancy). Gestational age (GA) was determined based on a first-trimester ultrasonographic examination or, in case it was not available, estimated from the date of the last menstrual period. New-born was examined immediately after bonding with a mother.

Average age was 31 ± 4 years and average BMI 24 ± 4. Table 1 presents the renal diagnoses with respective number of cases and characteristics of groups. Pre-pregnancy serum creatinine level (μmol/l) and proteinuria (g/l) is presented in Table 2.

**Table 1:** Patient description

Group no.	Kidney disease	Number of patients	BMI (mean)	BMI (SD	Age (mean)	Age (SD)
1	LN – lupus nephritis	17	23.6	3.2	29.7	3.4
2	IgA GN (Glomerulonephritis)	15	21.6	1.2	33.7	3.3
3	Other GN	16	23.9	6.9	29.6	4.4
4	CTIN (Chronic tubulointerstitial nephritis)	9	26.0	5.1	30.6	4.3
5	Congenital renal disease	9	23.4	2.2	30.0	3.8
6	Hypertensive nephrosclerosis	7	30.1	7.0	31.4	3.6
7	Diabetic nephropathy	3	24.0	4.6	35.3	5.1
8	History of kidney transplantation	1	20.0	–	27.0	–
9	Other	7	24.0	4.3	33.3	4.0
–	Summary	84	24.0	4.0	31.1	4.2

**Table 2:** Creatinine and proteinuria levels

Group n	Kidney disease	Number of patients	Creatinine pre pregnancy (mean)	Creatinine pre pregnancy (SD)	Proteinuria pre pregnancy (mean)	Proteinuria pre pregnancy (SD)
1	LN–lupus nephritis	17	92.2	45.1	1.13	1.31
2	IgA GN (Glomerulonephritis)	15	105.1	31.2	1.17	0.85
3	Other GN	16	68.4	16.8	1.10	1.03
4	CTIN (Chronic tubulointerstitial nephritis)	9	72.6	16.2	0.17	0.13
5	Congenital renal disease	9	92.3	43.2	0.03	0.01
6	Hypertensive nephrosclerosis	7	105.0	70.0	0.61	0.74
7	Diabetic nephropathy	3	132.0	57.0	0.50	0.04
8	History of kidney transplantation	1	121.0	–	0.03	0.00
9	Other	7	68.3	2.6	0.51	0.66
-	Summary	84	88.7	35.7	0.75	0.88

Normality of the data was tested using Shapiro–Wilk normality test, which excluded normal distribution (*p* < 0.001). To study the correlations between the gestation period, birth weight, creatinine levels and proteinuria we have used Spearman rank correlation test with a linear regression model. One-sided alternative of the Spearman correlation test with the prediction of greater creatinine levels or proteinuria levels predisposing to lower birth weight and shorter gestation period was used in all cases. *P* values < 0.05 were considered statistically significant. All statistical analyses were carried out using the RStudio software (Version 1.1.463, R version 3.5.2).

## Ethics approval

All procedures performed in our study were in accordance with the ethical standards of the General university hospital in Prague Ethics committee and with the 1964 Helsinki declaration and its later amendments.

## Consent to participate

All patients signed informed consent regarding analysis and publication of collected anonymized data.

## Results

### Analysis of the relation between serum creatinine levels and gestational period and birth weight

**Figure 1:**
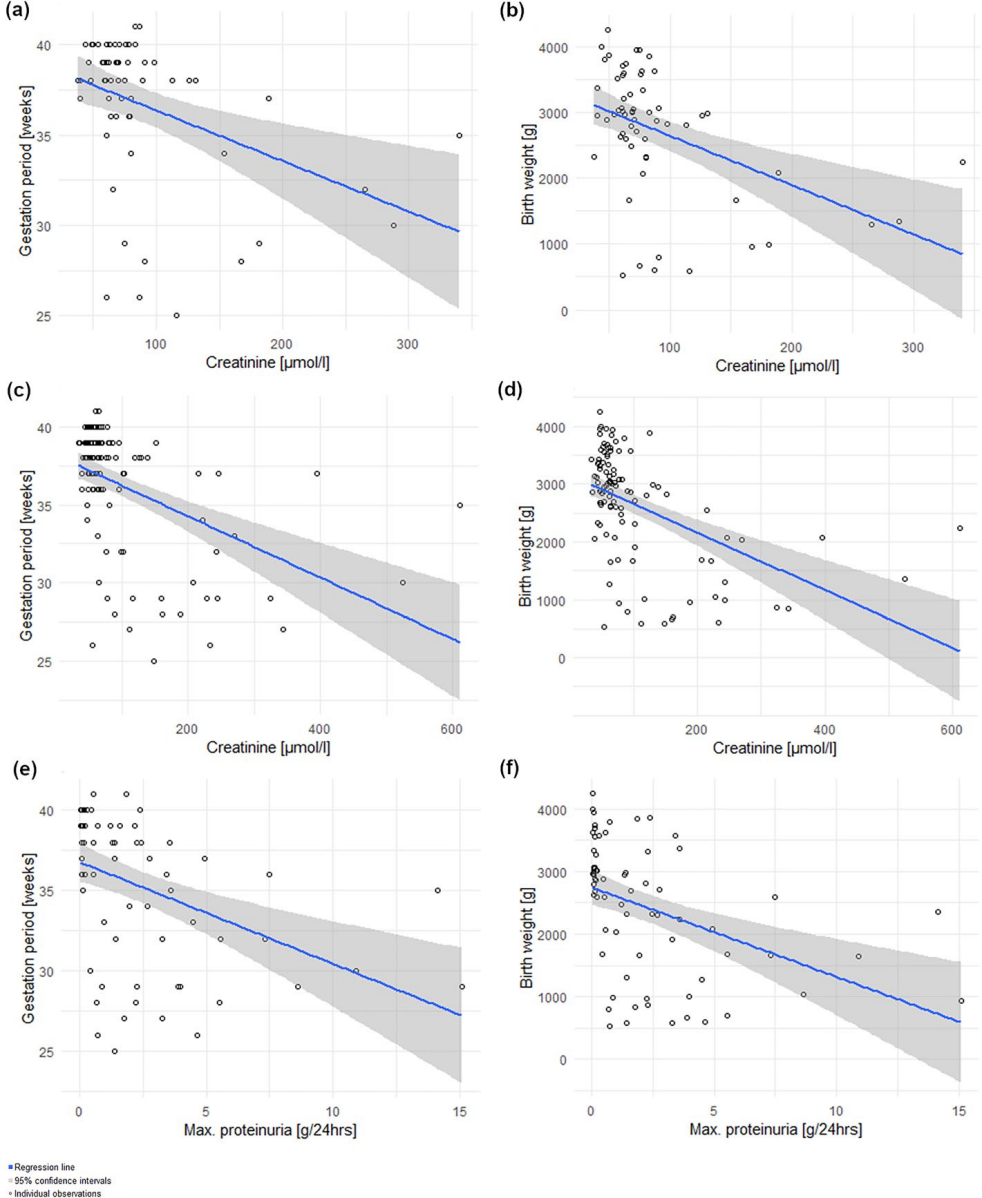
Creatinine and proteinuria analysis

The relationship between preconception serum creatinine value and gestation period is depicted in Fig. 1a. (Spearman’s rho = − 0.3692; *p* value = 0.002; R^2^ = 0.16). There was a negative correlation between the levels of preconception creatinine and the gestation period. The obtained *p* value of 0.002 was lower than the significance threshold of 0.05. Higher preconception creatinine levels were associated with shorter gestation period. The linear dependence explains 16% of the variability of the data as shown by the coefficient of determination of 0.16. Additional factors played a role in the length of the gestation period.

The relationship between preconception serum creatinine value and birth weight is depicted in Fig. 1b (Spearman’s rho = − 0.4571; *p* value < 0.001; R^2^ = 0.20). Negative correlation has been shown between the preconception creatinine levels and the birth weight. Higher preconception creatinine levels predisposed to a lower birth weight.

The relationship between maximum serum creatinine and gestation period (Spearman’s rho = − 0.4731; *p* value =  < 0.001; R^2^ = 0.22) and between maximum serum creatinine and birth weight (Spearman’s rho = − 0.5317, *p* value =  < 0.001, R^2^ = 0.24) is depicted in Fig. 1c, d.

Negative correlation has been shown for both gestation period and birth weight, thus higher maximum creatinine levels predisposed both to a shorter gestation period and a lower birth weight.

### Analysis of proteinuria

Influence of proteinuria on pregnancy outcomes was also analyzed. Figure 1e shows relationship between proteinuria and gestation period, while Fig. 1f shows relationship between proteinuria and birth weight. There was a negative correlation between proteinuria during gestation and the length of the gestation period (Spearman’s rho = − 0.5793; *p* value =  < 0.001; R^2^ = 0.18) and also between proteinuria and birth weight (Spearman’s rho = − 0.5863; *p* value =  < 0.001; R^2^ = 0.18). Higher proteinuria predisposed both to a shorter gestation period and lower birth weight.

### Analysis of max creatinine levels and birth weight in patients with lupus nephritis and IgA nephropathy

The analysis of two subgroups was done—lupus nephritis and IgA nephropathy.

**Figure 2:**
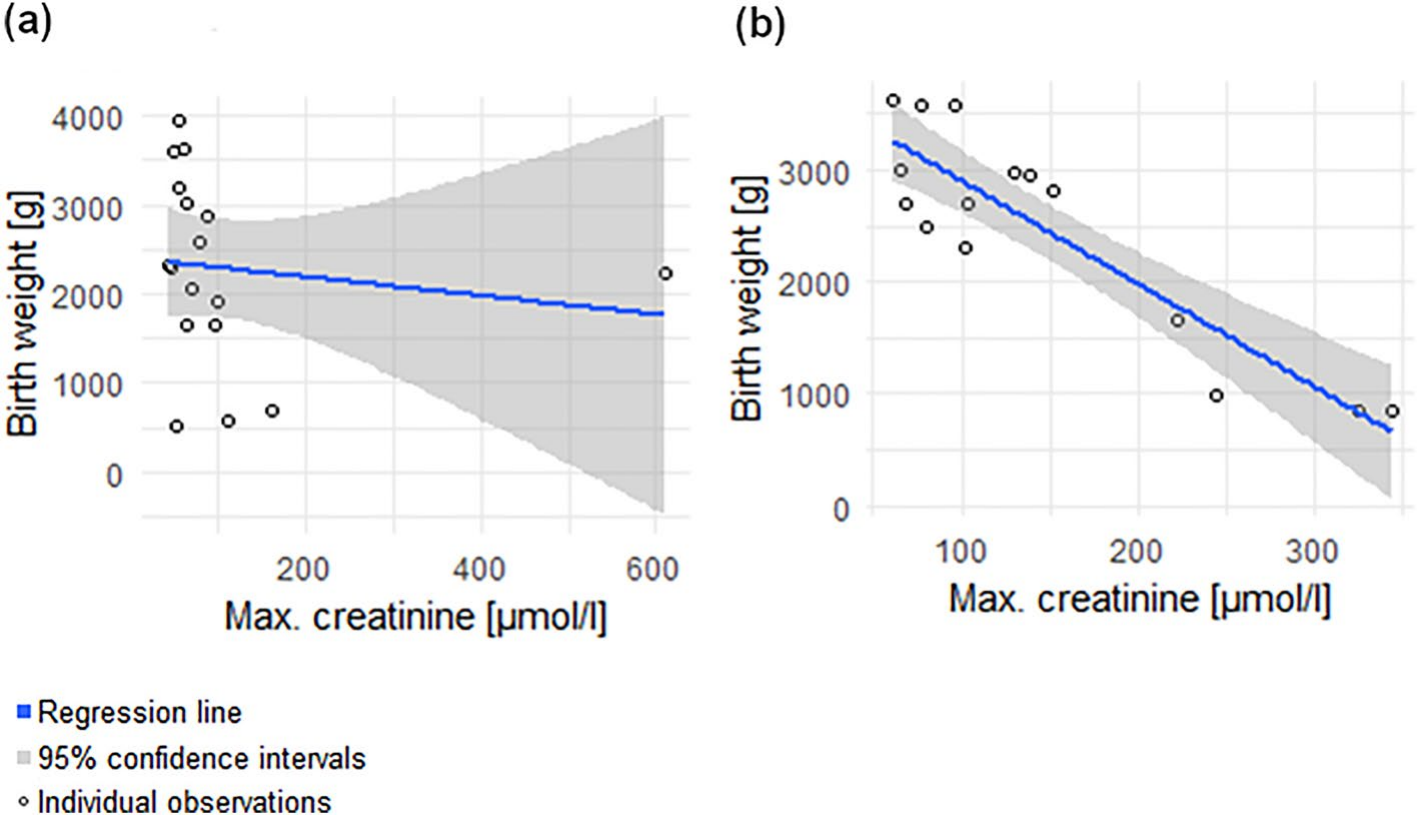
Subgroup analysis

The relationship between maximum serum creatinine and birth weight in lupus nephritis—group 1 (Spearman’s rho = − 0.4294; *p* value = 0.043) is depicted in Fig. 2a. The relationship between maximum serum creatinine and birth weight in IgA nephropathy – group 2 (Spearman’s rho = *-0.7393*, *p* value = 0.001) is depicted in Fig. 2b.

Negative correlation has been shown for both groups, thus higher maximum creatinine levels predisposed both to a lower birth weight. The correlation was much stronger in the subgroup of patients with IgA nephropathy.

### Pregnancy management

Patients were followed up on an outpatient basis, and care was under the guidance of a consultant in maternal–fetal medicine and a consultant in nephrology. Patients were admitted for hospitalization when there was progression of the underlying diagnosis, i.e., primarily uncorrected hypertension, worsening renal functions or signs of fetal compromise—especially fetal growth restriction. Predominating method of delivery in our CKD patients was caesarean section (64%). The main indications for termination of pregnancy in the study group were signs of maternal complications, mainly hypertension unresponsive to treatment, or rapid progression of renal dysfunction (for example creatinine elevation in a short period of time) or signs of fetal distress.

## Discussion

With advances in every field of medicine comes the need for new areas of expertise regarding pregnancies in high-risk patients. Feto-maternal medicine represents a field that demands constant interdisciplinary collaboration between obstetricians and other medical specialist. CKD encompasses a large non-homogenous group of diagnoses with which obstetricians commonly deal with during their clinical practice. Depending on when the diagnosis was made, CKD can be divided into two groups. The first and major group consists of patients with a preexisting condition, with diagnosis established before conception, and the second, minor group with patients whose condition first presented during the early stages of pregnancy. Regarding the first group, it is important to inform the patient of the risks and possible complications during pregnancy that can stem from and further worsen their CKD. Current scientific literature has very little data regarding how quantifiable and comparable maternal markers, such as creatinine and proteinuria, correspond with duration of pregnancy and fetal birth weight.

We have analyzed pregnancies of patients with chronic kidney disease diagnosed already before the conception. Adverse outcomes of pregnancies of patients with CKD have already been published in the literature—higher risk of preterm delivery, lower birth weight and higher number of admissions to neonatal intensive care unit have been reported^9^. In patients with IgA nephropathy, Piccoli et al. reported higher risk of preterm delivery but no risk of progression of kidney disease^10^. Blom et al. reviews patients with glomerulopathies and describes higher incidence of preterm delivery and lower birth weight^3^. Higher preconception and maximal creatinine during pregnancy led to a lower birth weight and shorter gestation period in our group of CKD patients. Findings from study by Piccoli et al. suggest an increased baseline risk for adverse pregnancy-related outcomes linked to CKD in all stages of CKD^11^.

Proteinuria in pregnant patients with IgA nephropathy is a significant risk factor for adverse pregnancy outcome^12,13^. This has been confirmed in our study—higher level of proteinuria led to shorter gestation period and lower birth weight.

In our study we demonstrated the correlation between creatinine levels and proteinuria as markers of kidney damage and pregnancy outcomes in terms of birth weight and length of gestation period are presented in our study. Risk of adverse pregnancy outcome in pregnant women with CKD is established. Proteinuria and higher stage of CKD is considered as significant risk factor for adverse pregnancy outcome^14^. Our study is confirming these data and presents new information on correlation between serum creatinine level, birth weight and pregnancy length before conception and during pregnancy. In larger scale, our study could help to predict pregnancy outcomes in patients with CKD based on renal function parameters. Limitation of our study is retrospective design, wide spectrum of underlying kidney disease and single-center data source. We are also fully aware of the inhomogeneous cohort, which includes a variety of diagnoses, some of which affect patients at older ages (nephroangiosclerosis). On the other hand, all the reported nephropathies have very similar effects accompanied by glomerular filtration disorders during otherwise physiological changes of pregnancy.

Increased risk of FGR in pregnant patients with CKD is questionable. Kendrick et al. and Piccolli et al. do not report increased risk of FGR, however the study by Zhang et al. presented higher risk of small for gestational age (SGA)^9,10,15^. Risk of stillborn is not increased in CKD patients^11^.

Chronic kidney disease and preeclampsia may both present with hypertension and proteinuria in pregnancy and they are often hard to distinguish. Uteroplacental flows and maternal circulating angiogenic factors like soluble fms-like tyrosine kinase 1 (sFlt-1), placental growth factor (PlGF) and their ratio are promising methods to distinguish between CKD and preeclampsia^16^. It is now acknowledged that preeclampsia can affect kidney health in the long term. Recent meta-analysis by Covella et al. showed that preeclampsia significantly increases the risk of end stage renal disease. However, there is lack of sufficient data to show a relationship between preeclampsia, albuminuria and chronic kidney disease. There is a need for further prospective studies in this area. Renal functions of every patient with preeclampsia should be followed at least for 12 weeks after the delivery, as the symptoms of preeclampsia should disappear at this time^17^. It is complicated to give a nomenclature to the situation in this field. All the patients we studied had preexisting kidney disease. It is virtually impossible to distinguish with certainty whether, in the case of worsening proteinuria or progression of creatinine elevation, the diagnosis should be labelled as preeclampsia or progression of the underlying kidney disease without the use of sflt-1/PlGF. The literature consensus of the authors is that currently the assessment of sflt-1/PlGF levels may help to differentiate the two situations, where a negative ratio may indicate the absence of the development of preeclampsia^18^. Research in this field points to the possibility of using the sflt-1/PlGF ratio to distinguish pre-eclampsia from active lupus nephritis^19^. The sflt-1/PlGF ratio can be used to differentiate between preeclampsia and nephropathy and this laboratory method is now routinely used to discriminate severe endothelial dysfunction accompanying possible preeclampsia. As our study group was established before the use of sflt-1/PlGF, this method was not included into the analysis.

Even though a significant improvement in the care for pregnant patients with CKD has been achieved in the recent years, our ability to predict and assess the safety of the pregnancy in these patients is still limited. Tools to identify CKD patients in high risk of adverse pregnancy outcomes should be focus of further research.

## Conclusion

Women of fertile age who were unable to conceive or carry a viable pregnancy just a short time ago now have that chance with the advancement of modern medicine. General population expects that those advances will also make it possible to carry a healthy child to term. Therefore, the aim of our study was to analyze the correlation of measurable markers in pregnant patients with adverse pregnancy outcomes. The risks of preterm delivery and lower birth weight of the neonate have been both demonstrated to be higher in patients with CKD. Higher serum creatinine levels (preconception and maximum during the gestation period) and higher proteinuria predispose to shorter gestation period and lower birth weight of the neonate. We believe that our work can provide new information as the results show that easily reproducible indicators—creatinine and proteinuria assessment—can be used in preconceptional consultation and during prenatal care, regardless of the type of kidney disease.

## Data availability

The datasets generated and analyzed during the current study are available from the corresponding author on reasonable request.
