# Author Correction: Chronic kidney disease and pregnancy outcomes

**DOI:** 10.1038/s41598-021-04142-6

**Published:** 2021-12-30

**Authors:** Jan Dvořák, Michal Koucký, Eva Jančová, Marek Mysliveček, Vladimír Tesař, Antonín Pařízek

**Affiliations:** 1 Department of Obstetrics and Gynaecology, General University Hospital in Prague and First Faculty of Medicine, Charles University, Apolinářská 18, 128 08 Prague 2, Czech Republic; 2 Department of Nephrology, General University Hospital in Prague and First Faculty of Medicine, Charles University, U Nemocnice 499/2, 128 08 Prague 2, Czech Republic

Correction to: *Scientific Reports* 10.1038/s41598-021-00670-3, published online 29 October 2021

The original version of this Article contained errors in the spelling of the authors Jan Dvořák, Michal Koucký, Eva Jančová, Marek Mysliveček, Vladimír Tesař and Antonín Pařízek, which were incorrectly given as Dvořák Jan, Koucký Michal, Jančová Eva, Mysliveček Marek, Tesař Vladimír and Pařízek Antonín.

The Author contribution statement now reads:

“J.D. – Main manuscript author – split authorship M.K. – Main manuscript author – split authorship E.J. – data collection M.M. – manuscript revision V.T.– manuscript revision A.P.- manuscript revision. All authors revised manuscript.”

The original Article has been corrected.

